# Genomic context as well as sequence of both *psr* and penicillin-binding protein 5 contributes to β-lactam resistance in *Enterococcus faecium*

**DOI:** 10.1128/mbio.00170-24

**Published:** 2024-04-02

**Authors:** Kavindra V. Singh, Jessica Galloway-Peña, Maria Camila Montealegre, Xingxing Dong, Barbara E. Murray

**Affiliations:** 1 Division of Infectious Diseases, Department of Internal Medicine, University of Texas Health Science Center, Houston, Texas, USA; 2 Department of Microbiology and Infectious Diseases, University of Texas Health Science Center, Houston, Texas, USA; University of Rochester, Rochester, New York, USA

**Keywords:** PBP5, Psr, ampicillin resistance, *E. faecium*

## Abstract

Penicillin-binding protein 5 (PBP5) of *Enterococcus faecium* (*Efm*) is vital for ampicillin resistance (AMP-R). We previously designated three forms of PBP5, namely, PBP5-S in *Efm* clade B strains [ampicillin susceptible (AMP-S)], PBP5-S/R (AMP-S or R), and PBP5-R (AMP-R) in clade A strains. Here, *pbp5* deletion resulted in a marked reduction in AMP minimum inhibitory concentrations (MICs) to 0.01–0.09 µg/mL for clade B and 0.12–0.19 µg/mL for clade A strains; *in situ* complementation restored parental AMP MICs. Using D344SRF (lacking *ftsW/psr/pbp5*), constructs with *ftsW*_A_*/psr*_A_ (from a clade A1 strain) cloned upstream of *pbp5-S* and *pbp5-S/R* alleles resulted in modest increases in MICs to 3–8 µg/mL, while high MICs (>64 µg/mL) were seen using *pbp5* from A1 strains. Next, using *ftsW ± psr* from clade B and clade A/B and B/A hybrid constructs, the presence of *psr*_B_, even alone or in *trans*, resulted in much lower AMP MICs (3–8 µg/mL) than when *psr*_A_ was present (MICs >64 µg/mL). qRT PCR showed relatively greater *pbp5* expression (*P* = 0.007) with *pbp5* cloned downstream of clade A1 *ftsW/psr* (MIC >128 µg/mL) vs when cloned downstream of clade B *ftsW/psr* (MIC 4–16 µg/mL), consistent with results in western blots. In conclusion, we report the effect of clade A vs B *psr* on AMP MICs as well as the impact of *pbp5* alleles from different clades. While previously, Psr was not thought to contribute to AMP MICs in *Efm,* our results showed that the presence of *psr_B_* resulted in a major decrease in *Efm* AMP MICs.

**IMPORTANCE:**

The findings of this study shed light on ampicillin resistance in *Enterococcus faecium* clade A strains. They underscore the significance of alterations in the amino acid sequence of penicillin-binding protein 5 (PBP5) and the pivotal role of the psr region in PBP5 expression and ampicillin resistance. Notably, the presence of a full-length psrB leads to reduced PBP5 expression and lower minimum inhibitory concentrations (MICs) of ampicillin compared to the presence of a shorter psrA, regardless of the pbp5 allele involved. Additionally, clade B *E. faecium* strains exhibit lower AMP MICs when both *psr* alleles from clades A and B are present, although it is important to consider other distinctions between clade A and B strains that may contribute to this effect. It is intriguing to note that the divergence between clade A and clade B *E. faecium* and the subsequent evolution of heightened AMP MICs in hospital-associated strains appear to coincide with changes in *Pbp5* and *psr*. These changes in *psr* may have resulted in an inactive Psr, facilitating increased PBP5 expression and greater ampicillin resistance. These results raise the possibility that a mimicker of PsrB, if one could be designed, might be able to lower MICs of ampicillin-resistant *E. faecium*, thus potentially resorting ampicillin to our therapeutic armamentarium for this species.

## INTRODUCTION

Enterococci are the second most common organism found in healthcare-associated (HA) infections, accounting for ~10%–15% of bacteria isolated from patients with these infections (1–3). Approximately, 80%–90% of clinical *Enterococcus faecium* (*Efm*) isolates are resistant to ampicillin (AMP-R) (4). When *Efm* strains are both AMP-R and vancomycin resistant, the two traditional drugs of choice, those infections can be very difficult to treat. Resistance to newer agents, such as linezolid and daptomycin, is also well documented and increasingly reported (5–7).

We now know that most clinical *Efm* isolates belong to a distinct *Efm* clade (clade A) that is evolutionarily separated by thousands of years from the community-associated (CA) clade B, comprising mostly human commensal isolates (8–10). Phenotypically, CA clade B is typically ampicillin susceptible (AMP-S) (MICs ranging from 0.125 to 2 µg/mL), while HA isolates are usually AMP-R (MICs often >64 µg/mL) (8, 11). Penicillin-binding protein 5 (PBP5) from AMP-R strains of *Efm* is a low-affinity penicillin-binding protein that has been shown to be essential for AMP-R (12, 13). There are two major and distinct PBP5 forms: PBP5-S, which is found in AMP-S CA clade B *Efm*, and PBP5-R, which is found in AMP-R HA clade A *Efm* (8, 11). We have previously classified another form, PBP5-S/R, which appears to be a transitional form of PBP5 that is found in what has been designated (sub)clade A2 (closely related to clade A1 and commonly found in animals) (14). The *pbp5-R* and *pbp5-S* alleles differ by ~5% in their nucleotide sequence, and we previously reported a consensus of 20–21 amino acids (aa) (some PBP5-Rs have an additional aa) that can distinguish -S from -R proteins (11, 14). A combination of four aa changes, made in PBP5-S/R from an AMP-S, (sub)clade A2 strain, was able to decrease the affinity of the resulting recombinant PBP5 protein for penicillin by 16-fold and increased the AMP MIC 5-fold (15). The generally accepted notion is that the intense pressure of β-lactam use in hospitals has selected for progressively more resistant strains via mutations in *pbp5-S/R*, resulting in more of the “R-specific” residues. Although we observed that hybrid PBP5-S/R patterns show a progression of amino acid changes from the S form to the R form of this protein, we could not strictly correlate these changes with ampicillin MICs (11, 14).

Not only is the amino acid sequence of PBP5 important, but it appears that greater expression of *Efm* PBP5 is also important in mediating ampicillin resistance (16), yet it is still poorly understood. An early study of an AMP-R *Efm* strain (C68) found a homolog of *psr* from *E. hirae* (originally named as “**P**BP **s**ynthesis **r**epressor”) directly upstream of *pbp5* (12). In *E. hirae*, the *psr* gene upstream of its *pbp5* gene was concluded to contribute to penicillin resistance levels since an 87-bp deletion in the 5′ region of the *psr* ORF was associated with increased PBP5 levels and penicillin resistance: this led to the designation of this ORF as a repressor of *pbp5* expression (17). In *Efm*, however, a role for Psr in *pbp5* expression is not established. Rice et al. reported that Psr of the AMP-R HA strain C68 had an early stop codon, concluding that Psr was inactive and proposed that it did not participate in the regulation of *pbp5* transcription or ampicillin resistance (12).

In our studies, to help delineate the role of *Efm* Psr, we previously reported that *psr* in HA clade A AMP-R *Efm* (*psr*_A_) is truncated relative to *psr* in commensal AMP-S *Efm* (*psr*_B_) (16). Specifically, high-ampicillin MICs were seen in strains with a 201-bp deletion in *psr*, consistent with the report mentioned above with *E. hirae*. This deletion encompasses 137 bp of the 3′ end of *psr* and 64 bp of the intergenic region of *psr* and *pbp5*. There are variations in deletions, and in some cases, there are insertions in the *psr* region in strains belonging to A1 or A2 cladal groups showing slight variations in high AMP MICs. Strain C68, as mentioned above (12), also has an insertion of one nucleotide in the *psr*-coding sequence resulting in an early stop codon (12), in addition to the missing downstream 201 bp. Thus, the presence of the early stop codon would likely not have any additional effect vs the effect of truncation.

Herein, we sought (i) to further explore the contributions of *pbp5* sequence and clade background to AMP-R and (ii) to address our hypothesis that the presence of the longer (“full length”) Psr seen in commensal AMP-S *Efm* (i.e., *psr*_B_) leads to lower AMP MICs and repression of PBP5 expression relative to the presence of truncated *psr* typically found in HA strains. To test this hypothesis, we performed functional studies by cloning different *pbp5* alleles downstream of various native or hybrid *psr* or *ftsW/psr* regions and ascertained the effects on AMP MICs and *pbp5* expression.

## MATERIALS AND METHODS

### Bacterial strains, plasmids, routine growth conditions, and susceptibility testing

Relevant characteristics of all *Efm* and *Escherichia coli* strains, newly created plasmid vectors, and *pbp5*, *psr,* and *ftsW* constructs used in the current study are described in Table S1; clinical *Efm* isolates and their AMP MICs are also described in Tables 1 to 4, while Fig. S1 shows a schematic representation of the *pbp5* and the surrounding genes of clade B *E. faecium* strains COM15 and subclade A1 C68 (16). *Efm* D344SRF (18) was used as the host strain for all the plasmid constructs used in this study and is a derivative of a healthcare-associated (clade A1, original AMP MIC 24 µg/mL) clinical *Efm* that became AMP susceptible (MIC 0.023 µg/mL) following a 160-kb spontaneous genomic deletion that included *pbp5*, *ftsW,* and *psr* (18). pCWR plasmids were kindly provided by LB Rice and are described below and in footnotes of Tables 2 to 4 and Table S1. Naturally occurring *pbp5-S, pbp5-S/R* “hybrid,” and *pbp5-R* alleles encoding PBP5-S, PBP5 S/R, and PBP5-R and their previously published consensus amino acids residue profiles are described when used in the tables as well as Table S1; in this nomenclature, S indicates an amino acid that is part of the consensus sequence of 20–21 aa characteristic of AMP-S clade B strains, and R indicates an amino acid that is part of the consensus sequence characteristic of AMP-R clade A strains (11, 14, 19). Among these, the *pbp5* alleles studied here came from two clade B *Efm* strains COM15 (S21/R0) (10, 14, 20) and E980 (S16/R5) (14, 20, 21) (referred to as *pbp5*_S_ and PBP5-S); two clade A2 *Efm* strains D366 (S7/R14) (11, 14) and E1679 (S3/R18) (21) (*pbp5_S/R_* encoding PBP5-S/R); and three clade A1 *Efm* strains C68 (S1/R20) (16, 22), 1.231.502 (S1/R20) (10, 16), and TX82 (S0/R21) (16, 23) (*pbp5_R_* encoding PBP5-R).

**TABLE 1:** Origin and AMP MICs of WT strains, *pbp5* deletion mutants, and derivatives with the WT *pbp5* reconstituted in its native chromosomal location

Bacteria and constructs used (PBP5 amino acid profile^*a*^ and clade)	AMP MICs (µg/mL)	References
*E. faecium* COM15^*b*^, fecal isolate from healthy volunteer (S21/R0^*a*^, clade B)	0.12–0.19	(10, 14, 20)
COM15:Δ*pbp5*	0.064	This study
*E. faecium* TX1330, fecal isolate from healthy volunteer (S21/R0, clade B)	1–1.5	(16, 20)
TX1330:Δ*pbp5*	0.01–0.09	This study
TX1330:Δ*pbp5::pbp5*_TX1330_ reconstituted *in situ* in the chromosome	1.5	This study
*E. faecium* E1162 bloodstream isolate (S1/R20, clade A1)	24–32	(24)
E1162:Δ*pbp5*	0.12–0.19	(24)
E1162:Δ*pbp5::pbp5*_E1162_ reconstituted *in situ* in the chromosome	32–48	This study

^*a*^ Number of amino acids characteristic of PBP5-S consensus (S)/number of aa characteristic of PBP5-R consensus -R (11, 14).

^*b*^ COM15 was previously shown to produce less PBP5 than TX1330 via western blots (16).

**TABLE 2:** AMP-MICs conferred by *pbp5* alleles from different *E. faecium* clades cloned in *trans* into the same *pbp5*-lacking background^*e*^

Strain origin of cloned *pbp5* alleles (PBP5 amino acid profile^*a*^ and clade)	AMP MICs (µg/mL)	
	Wild-type *E. faecium*	D344SRF^*c*^ ± pCWR620^*b*^^,^^*c*^ containing the *pbp5* allele from the WT strains in the first column
D344SRFΔ*pbp5* (NA, A1)^*c*^	0.023^*d*^	NA
TX82 *pbp5-R* (S0/R21, A1)	64	128–256
C68 *pbp5-R* (S1/R20, A1)	128	64–128
1.231.502 *pbp5-R* (S1/R20, A1)	128/>256	128–256
E1679 *pbp5-R* (S3/R18, A2)	256	192
D366 *pbp5-R/* S (S7/R14, A2)	1.5	6
E980 *pbp5-S* (S16/R5, B)	0.75–1	6–8
COM15 *pbp5-S* (S21/R0, B)	0.12–0.19	3–4

^*a*^ Number of amino acids characteristic of PBP5-S consensus (S)/number of aa characteristics of PBP5-R consensus -R (11, 14).

^*b*^ pCWR620 is a published vector (15) derived from the shuttle vector pTCV-lac and contains *ftsW* and *psr* from the clade A1 *E. faecium* strain C68; *psr* of C68 has a 201-bp deletion and a premature stop codon relative to *psr* from clade B strains (15, 16).

^*c*^ Host strain D344SRFΔ*ftsW*Δ*psr*Δ*pbp5* used for all plasmid constructs is a healthcare-associated (clade A1) *E. faecium* and that has a 160-kb spontaneous genomic deletion encompassing *pbp5*, *ftsW*, *psr* regions (15, 18).

^*d*^ NA, not applicable (see ^*c*^).

^*e*^ NA, not applicable since this strain has no *pbp*5 to clone.

**TABLE 3:** Effect of *ftsW/psr* from clade A and clade B on AMP MICs conferred by *pbp5* alleles cloned from different *E. faecium* clades into the same *pbp5*-lacking background^*f*^

Strain origin of cloned *pbp5* alleles (aa profile^*a*^ and clade) [AMP MIC (µg/mL)]	AMP MICs (µg/mL)	
	*pbp5* allele from first column cloned downstream of clade A1 *ftsW*_C68_/*psr*_C68_^*b*^	*pbp5* allele from first column cloned downstream of clade B *ftsW*_COM15_/*psr*_COM15_^*c*^
D344SRFΔ*pbp5* (A1)^*d*^ (0.023)	NA	NA
TX82 *pbp5* (S0/R21, A1) (64)	128–256	4–6
C68 *pbp5-R* (S1/R20, A1) (128)	64–128^*e*^	4
1.231.502 *pbp5* (S1/R20, A1) (128–≥256)	128–256	8–12
E1679 *pbp5* (S3/R18, A2) (256)	192	8–16
D366 *pbp5* (S7/R14, A2) (1.5)	6	3
E980 *pbp5* (S16/R5, B) (0.75–1)	6–8	2–3
Com15 *pbp5* (S21/R0, B) (0.12–0.19)	3–4	0.5–0.75

^*a*^ Number of amino acids characteristic of PBP5-S consensus (S)/number of aa characteristics of PBP5-R consensus -R (11, 14).

^*b*^ pCWR620 is a published vector (15) derived from the shuttle vector pTCV-lac and contains *ftsW* and *psr* from the clade A1 *E. faecium* strain C68; *psr* of C68 has a 201-bp deletion and a premature stop codon relative to *psr* from clade B strains.

^*c*^ pTEX6162 (this study) is derived from the shuttle vector pTCV-lac and contains *ftsW*_B_ and *psr*_B_ from the AMP-S clade B *E. faecium* strain COM15; this *psr* does not have the 201-bp deletion or the premature stop codon seen in *psr* of C68 (15, 16).

^*d*^ Host strain D344SRF used for all plasmid constructs is a healthcare-associated (clade A1) *E. faecium* that has a 160-kb spontaneous deletion encompassing *pbp5*, *ftsW*, *psr* regions (16).

^*e*^ Range of MICs reflects results with different colonies and/or repetitions.

^*f*^ NA, not applicable since this strain has no *pbp*5.

**TABLE 4:** Effect of *psr* alone and of hybrid *ftsW/psr* constructs from an AMP-S clade B strain and an AMP-R clade A strain on AMP MICs generated by *pbp5* alleles cloned from different *E. faecium* clades in the same *pbp5*-lacking background^*a*^

Strain origin of cloned *pbp5* alleles (aa profile^*b*^ and clade) (AMP MIC (µg/mL)	AMP MICs (µg/mL)			
	*pbp5* alleles cloned downstream of clade A1 *psr*_A1_^*c*^	*pbp5* alleles cloned downstream of clade B *psr*_B_^*d*^	*pbp5* alleles cloned downstream of hybrid *ftsW*_A1_/*psr*_B_^*e*^	*pbp5* alleles cloned downstream of hybrid *ftsW*_B_/*psr*_A1_^*f*^
TX82 *pbp5* (S0/R21, A1) (64)	32–64^*g*^	8	8	24
C68 *pbp5* (S1/R20, A1) (128)	32–64	2–4	8	48–64
1.231.502 *pbp5* (S1/R20, A1) (128/>256)	256	16	8	256
E1679 *pbp5* (S3/R18, A2) (256)	Not done	Not done	6	24
D366 *pbp5* (S7/R14, A2) (1.5)	6	3	3	6–8
E980 *pbp5* (S16/R5, B) 0.75–1)	6–8	3–4	3–4	3
Com15 *pbp5* (S21/R0, B) (0.12–0.19)	4	1	1	3–4

^*a*^ Host strain D344SRF used for all plasmid constructs is a healthcare-associated (clade A1) *E. faecium* and has a 160-kb spontaneous genomic deletion encompassing *pbp5*, *ftsW*, *psr* regions (15).

^*b*^ Number of amino acids characteristic of PBP5-S consensus (S)/number of aa characteristics of PBP5-R consensus (R) (11, 14).

^*c*^ Host plasmid pTEX6172 (this study) is derived from the shuttle vector pTCV-lac and contains *psr*_A1_ from clade A1 *E. faecium* strain C68 which has a deletion of 201 bp relative to clade B *psr* alleles and a premature stop codon.

^*d*^ Host plasmid pTEX6173 (this study) is derived from the shuttle vector pTCV-lac and contains *psr*_B_ from *E. faecium* COM15 and does not have the 201-bp deletion or the premature stop codon found in *psr*_C68_.

^*e*^ Host plasmid pTEX6163 (this study) is derived from the shuttle vector pTCV-lac and contains *ftsW*_A1_ from AMP-R, clade A1 *E. faecium* C68, and *psr*_B_ was from AMP-S, clade B *E. faecium* COM15.

^*f*^ Host plasmid pTEX6164 (this study) is derived from the shuttle vector pTCV-lac and contains *ftsW*_B_ from AMP-S, clade B *E. faecium* strain COM15, and *psr*_A1_ was from AMP-R, clade A1 *E. faecium* C68.

^*g*^ Range of AMP MICs reflects results with different colonies and/or repetitions.

*Efm* strains were grown at 37°C in brain heart infusion (BHI) (Becton, Dickinson (BD), Franklin Lakes, NJ) broth or agar with or without antibiotics as needed. *E. coli* strains were grown at 37°C using Luria-Bertani (LB; BD) broth or agar with or without antibiotics. AMP susceptibility testing was performed by broth microdilution in Mueller Hinton II (MH II) broth (cation adjusted; BD) following the Clinical and Laboratory Standards Institute guidelines (25) or by *E*-test (bioMérieux, France) for WT *E. faecium* strains. In the case of D344SRF and its derivatives with and without *pbp5* plasmid constructs, MICs were performed on BHI agar via agar dilution method or with *E*-test since this strain grows very poorly on MH agar II. Kanamycin (KAN) and AMP were purchased from Sigma-Aldrich, St. Louis, MO.

### Generation of *pbp5* deletion mutants and *pbp5* reconstituted *in situ* in the chromosome

Previously published methods by our group (26–29) were followed to construct chromosomal gene deletion mutants and to generate their chromosomal reconstituted/complemented constructs. To construct chromosomal *pbp5-S* deletion mutants, we used AMP-S clade B *Efm* strains: COM15 (16), which produces very little PBP5 and TX1330, which produces slightly more PBP5 than COM15 (16). Genomic DNA regions flanking the *pbp5* gene from 982 nucleotides (nt) upstream (UP fragment) and 1,142 nt downstream (DW fragment) were amplified by overlap extension PCR using primers containing NotI and BamHI restriction sites (Table S1) and cloned into NotI and BamHI sites of plasmid pHOU1 (16). pHOU1 contains a pheS* allele, encoding a phenylalanine tRNA synthetase with altered substrate specificity, which confers susceptibility to *p*-chloro-phenylalanine (*p*-Cl-Phe) and has a gentamicin (GEN) resistance marker (26–29). The recombinant plasmid was then electroporated into the conjugative donor strain, *E. faecalis* CK111 (30), where it can replicate. The resulting donor strain was used in a conjugation experiment by filter mating with *Efm* strains COM15 and TX1330. Single-crossover integrants were selected on BHI plates containing gentamicin and erythromycin and then replated onto MM9YEG medium (M9-based medium supplemented with yeast extract, salts, and glucose) containing *p*-Cl-Phe (7 mM) to select for excision of pHOU1. We confirmed the excision of pHOU1 by the absence of growth on BHI + GEN 100–125 µg/mL agar plates, and colonies lacking *pbp5* were detected by PCR. The correct *pbp5* deletion was also confirmed by sequencing of the PCR fragment obtained by using outside primers specific for the flanking regions of the cloned gene and by pulsed-field gel electrophoresis to confirm the host identity following the methods we have previously described (26–29). The resulting *pbp5* deletion mutants COM15Δ*pbp5* and TX1330Δ*pbp5* were designated as TX6153 and TX6260, respectively (Table S1), and their AMP MICs were determined.

To restore the wild-type gene of TX1330Δ*pbp5* back into its native location in the chromosome, in brief, the *pbp5* gene of TX1330 along with the upstream and downstream sequences was amplified using overlapping primers (Table S2), which ensured the removal of the BamHI site present at both ends of *pbp5* gene, and then the above published methods were followed (26–29) to create TX6262 (TX1330:Δ*pbp5::pbp5*_TX1330_). The *pbp5* coding and promoter regions were sequenced to ensure that no unintended mutations had occurred, followed by determining the AMP MICs. We were unable to create a chromosomally complemented strain for COM15Δ*pbp5* as will be explained in the Results section.

For the deletion of *pbp5-R* from a clade A1 strain, we used *Efm* strain E1162 (S1/R20) and its previously published *pbp5* deletion mutant E1162Δ*pbp5,* which is a deletion of *pbp5* after the first ~500 bp of the *pbp5* gene (24). To complement E1162:Δ*pbp5,* we followed the methods above using the pHOU1 system (26–29) and a PCR-generated fragment encompassing 66 bp upstream of the *pbp5* start codon (including promoter region) plus 2,037 bp of the *pbp5*_E1162_-coding region plus 629 bp downstream of *pbp5* sequence. The resulting strain, E1162:Δ*pbp5::pbp5*_E1162_ (TX6261), was tested for AMP MICs.

### Construction and use of plasmid vectors for cloning various naturally occurring *pbp5* alleles

To study the phenotypes of naturally occurring *pbp5* alleles in D344SRF, we chose pCWR620 (kindly provided by Lou Rice) (15) which shows *pbp5* expression levels similar to WT *pbp5* for *Efm* strain C68 (16, 22) (Table S1). pCWR620 was originally designed to facilitate expression of *pbp5-R* from an AMP-R clade A1 *Efm* C68 cloned into an AMP-susceptible *Efm* strain D344SRF, which, as mentioned, lacks *pbp5* (15). pCWR620 [also designated here pCWR620 (*ftsW*_C68_/*psr*_C68_)] contains *ftsW* and *psr* from *Efm* strain C68, from upstream of the *ftsW* promoter to downstream of the *pbp5* promoter, inserted into the shuttle vector pTCV-lac (15, 31). As we previously reported, C68 *psr* is truncated in pCWR620, relative to *psr* from clade B strains (16). In addition, it has a mutation in the coding region resulting in an early stop codon and is thought to be non-functional (15). To study various *pbp5* alleles (*S, S/R,* and *R*) in this vector, we first PCR amplified *pbp5* from different *Efm* strains, namely, two clade B *Efm* COM15 (S21/R0) and E980 (S16/R5); two clade A2 *Efm* D366 (S7/R14) and E1679 (S3/R18); three clade A1 *Efm* strains C68 (S1/R20), 1.231.502 (S1/R20), and TX82 (S0/R21). Designed PCR primers had BamHI restriction sites inserted on both ends which were used to clone amplified *pbp5* fragments into pCWR620 (*ftsW*_C68_/*psr*_C68_), followed by the transformation into *E. coli* DH5′ with selection on LB agar plates supplemented with kanamycin 50 µg/mL. After confirming the insert size and the nucleotide sequence, the recombinant plasmid DNA was electroporated into AMP-susceptible D344SRF lacking *pbp5* with selection on Todd Hewitt agar plates containing 0.25M sucrose plus KAN 1,500 µg/mL, and resulting constructs were saved and are listed in Table S1.

The construction of *ftsW_A1_/psr_A_*_1_ and *ftsW_B_/psr_B_* plasmids in pTCV-lac involved the use of two of our *Efm* strains, i.e., C68 (subclade A1) and COM15 (clade B), respectively. The configuration of their *pbp5* and surrounding genes is illustrated in Fig. S1, sourced from our previously published paper (16).

**Fig 1:**
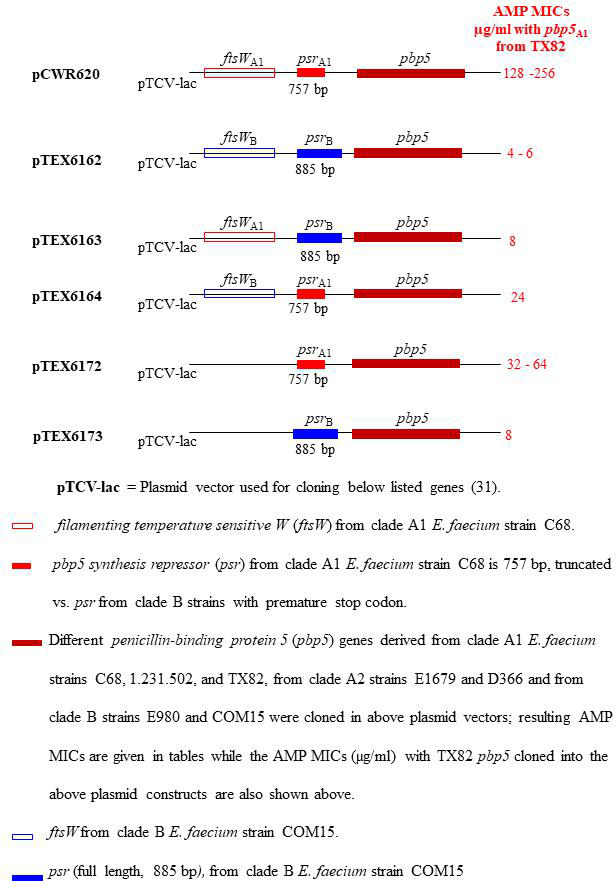
Illustrative depiction of the plasmid vectors, derived from pTCV-lac, for investigating effects of different *pbp5, ftsW, and psr* alleles. The expression vector pTCV-lac was employed for cloning *ftsW and psr* alleles from clade A1 (C68) and clade B (COM15) *E. faecium* strains, as well as through cross-pairing (hybrid) of *ftsW* and *psr* from these clades, at the SmaI-BamHI site. The *ftsW/psr* segments are visually represented in blue (COM15) and red (C68). The host strain, D344SRF, is a healthcare-associated clade A1 *E. faecium* with a prior spontaneous genomic deletion spanning 160 kb, which includes the *pbp5, ftsW*, and *psr* regions. pCWR620 (15)-*ftsW*_A1_*/psr*_A1_ from clade A1 strain C68 exhibits a naturally truncated *psr* and has a mutation in the coding region, leading to the presence of an early stop codon. pTEX6162-*ftsW*_B_*/psr*_B_ is from COM15; it has full length *psr*. pTEX6163-*ftsW*_A1_ is from C68, and *psr*_B_ (full length) is from COM15. pTEX6164-*ftsW*_B_ is from COM15, and *psr*_A1_ (truncated with premature stop codon) is from C68. pTEX6172-*psr*_A1_ (truncated with premature stop codon) is from C68. pTEX6173-*psr*_B_ (full length) is from COM15.

As mentioned above, *psr* in pCWR620 (*ftsW*_C68_/*psr*_C68_) is thought to be non-functional (15); in addition, *psr* alleles from multiple clade A strains were noted previously to be consistently shorter than *psr* from clade B strains (16). To further explore possible differences associated with *psr* from different strains, we next created a vector with a *psr* allele with no apparent truncation or mutation along with *ftsW* from a clade B strain COM15 as discussed below. To create this new vector, we adopted the same strategy as described for construction of pCWR620 (*ftsW*_C68_/*psr*_C68_) (15). PCR-amplified *psr* and *ftsW* from clade B strain COM15 from upstream of the *ftsW* promoter to downstream of *psr* including the *pbp5* promoter (conserved among clade A1 and clade B strains studied) were obtained. Primers used for PCR amplification contained SmaI (upstream) and BamHI (downstream) restriction sites. The amplified *ftsW/psr* fragment was cloned into shuttle vector pTCV-lac at the SmaI/BamHI site. The resulting plasmid construct was designated as pTEX6162(*ftsW*_COM15_/*psr*_COM15_). We then used pTEX6162 to clone *pbp5* alleles from strains COM15 (S21/R0, clade B), E980 (S16/R5, clade B), D366 (S7/R14, clade A2), E1679 (S3/R18, clade A2), C68 (S1/R20, clade A1), 1.231.502 (S1/R20, clade A1), and TX82 (S0/R21, clade A1). Transformation of recombinant plasmids into *E. coli* DH5′ and subsequent electroporation into D344SRF was done (23) for pCWR620 (*ftsW*_C68_/*psr*_C68_) (15). Resulting plasmid constructs are listed in Table S1, and a schematic of pTEX6162(*ftsW*_COM15_/*psr*_COM15_) is provided in Fig. 1.

Based on the AMP MICs obtained with the various constructs above, we decided to create four additional vectors using the same strategy as described above for pTEX6162 (*ftsW*_COM15_/*psr*_COM15_) with a goal to study and compare AMP MICs of various *pbp5* alleles with clade A1 and clade B *psr*, with and without their corresponding *ftsW* as well as cross-pairing (hybrid) of *ftsW* and *psr* in newly created vectors. In order to achieve this, we first created two hybrid vectors where clade A1 *ftsW*_C68_ was joined with clade B *psr*_COM15_ and vice versa via crossover PCR followed by cloning into pTCV-lac to generate pTEX6163 (*ftsW*_C68_/*psr*_COM15_) and pTEX6164 (*ftsW*_COM15_/*psr*_C68_), respectively. Second, we amplified *psr* alone with its promoter region from clade A1 *E. faecium* C68 (*psr*_C68_) and clade B *E. faecium* COM15 (*psr*_COM15_) with BamHI restriction sites in both ends, followed by cloning at the BamHI site of pTCV-lac; the resulting plasmids were designated as pTEX6172 (*psr*_C68_) and pTEX6173 (*psr*_COM15_), respectively (Table S1). All of the above mentioned various *pbp5* alleles were cloned into pTCV-lac, resulting in pTEX6163 (*ftsW*_C68_/*psr*_COM15_), pTEX6164 (*ftsW*_COM15_/*psr*_C68_), pTEX6172 (*psr*_C68_), and pTEX6173 (*psr*_COM15_), followed by transformation into *E. coli* DH5′, extraction of recombinant plasmids, and then electroporation back into D344SRF. Resulting constructs are listed in Table S1 and Fig. 1, and their AMP MICs were determined and compared.

Additionally, we also analyzed two of the previously published plasmids, pCWR624 and pCWR666, carrying site-directed mutagenized *pbp5* of clade A1 origin (15) in some of our *pbp5* deletion mutants generated here, namely COM15Δ*pbp5* and TX1330Δ*pbp5*, both belonging to clade B, and E1162Δ*pbp5* belonging to clade A1 (24), to evaluate and compare their AMP MICs vs the WT parent *Efm* strains COM15, TX1330, and E1162, respectively. In pCWR624, amino acid residues at positions 485, 499, and 629 in the active site region of PBP5 are methionine, isoleucine, and glutamic acid (typical of PBP5-S); in pCWR666, amino acids typical of PBP5-R are present at these sites, namely alanine, threonine, and valine, respectively, in addition to having serine at 466 position which is absent in pCWR624 (Table S1). The AMP MICs of these constructs in D344SRF have been reported earlier as 38 and 185 µg/mL, respectively (15).

### Quantitative real-time PCR

For qRT-PCR analysis, three biological replicates per each test strain were used, and for each biological replicate, three technical replicates were used. We selected three representative constructs as described here to determine *pbp5* expression levels. *pbp5* derived from *Efm* TX82 (S0/R21) and cloned into pCWR620 (*ftsW*_C68_/*psr*_C68_), i.e., TX6255 [D344SRF (pCWR620::*pbp5*_S0/R21_TX82_)] and in pTEX6162 (*ftsW*_COM15_/*psr*_COM15_), i.e., TX6227 [D344SRF (pTEX6162::*pbp5*_S0/R21_TX82_)] was used. Control AMP-susceptible *Efm* strain D344SRF carrying the empty pTCV-lac vector was also used for baseline and for statistical significance. Bacteria were regrown from an overnight culture at 37°C with gentle agitation until an 0.8 OD_600_, and then cells were harvested. RNA extraction was done following standard methods (32) followed by synthesis of complementary DNA (cDNA) and evaluation of gene expression with 10 ng cDNA using SYBR Green (32). The *gyrB_Efm_* housekeeping gene (Table S2) was used for normalization. The intragenic *pbp5* gene prime pair (Table S2) was used to determine *pbp5* level of expression in all three test bacteria, and the primers were designed from TX82 *pbp5* DNA sequence. Differential gene expression was analyzed with an unpaired (two-tailed) *t* test, and *P* < 0.05 was considered significant.

NCBI website http://www.ncbi.nlm.nih.gov/genome was used to retrieve the *ftsW*, *psr,* or *pbp5* sequences from test bacteria used in the current study. DNA and protein multiple sequence alignments were performed using the alignment tool MUSCLE from the EBI website http://www.ebi.ac.uk/Tools/msa/muscle/ for sequence confirmation of cloned fragments. The *E. faecium* strains analyzed for FtsW alignment, along with their corresponding GenBank accession numbers, were COM15 (NZ_CP025022), E980 (NZ_ABQA01000020.1), TX1330 (NZ_QYBD01000001.1), 1.231.502 (NZ_GG688486.1), C68 (NZ_LRAQ01000090.1NZ_LRAQ01000090.1), TX16 (CP003583.1), E1162 (NZ_ABQJ01000055.1), TX82 (NZ_GL455914.1), and E1679 (NZ_ABSC01000284.1). The primers for PCR amplification or nucleotide sequencing confirmation are listed in Table S1.

### Generation of anti-rPBP5-R antibodies

Polyclonal antibodies against rPBP5-R (C68) were separately generated using a previously described scheme and as described earlier by us (16, 33, 34). Approved protocol and guidelines by the Animal Welfare Committee of The University of Texas Health Science Center, Houston, TX were followed.

### PBP5 detection by western blotting

Cells grown overnight in BHI broth were inoculated into fresh BHI broth at a starting optical density at 600 nm (OD600) of 0.05 and grown at 37°C with gentle shaking until they reached an OD_600_ of ~0.1–0.2. The cultures were centrifuged at 3,900 rpm for 10 min, and the pellets were rapidly chilled on dry ice. Cells were washed with 0.02 M Tris-HCl, pH 7.0, and 0.01 M MgSO_4_ buffer and resuspended in one-tenth the volume of the same buffer containing 100 µM PMSF. Following the addition of mutanolysin to a final concentration of 10 U/1 OD_600_ of cells, tubes were incubated at 37°C for 1 h in a rotating shaker, followed by centrifugation to recover the supernatant (16, 33). Protein concentrations were measured as described previously (16, 33). Samples were normalized so that the total protein concentrations were equally followed by mixing (1:1) in 2× Laemmli sample buffer (BIO RAD, CA). Samples were treated at 100°C for 10 min prior to separating them by 4%–20% Mini-PROTEAN TGX Precast Protein Gels (BIO RAD, CA) under reducing conditions in running buffer and transferred to a nitrocellulose membrane following the manufacturer’s protocol. Membranes were then probed with rPBP5-R (C68) polyclonal rat sera preabsorbed for 2 h with cell lysates of D344SRF in order to remove non-specific background bands, followed by HRP-conjugated goat anti-rat IgG antibodies (secondary antibody) and developed using Bio-Rad’s chemiluminescent detection reagents (BIO RAD, CA) per manufacturer’s instructions.

## RESULTS

### AMP susceptibility testing of *pbp5* deletion mutants and their derivatives with *pbp5* reconstituted *in situ* in the chromosome

The construct details and AMP MICs are shown in Table 1. Deletion of *pbp5* from the clade B *E. faecium* strain COM15 resulted in only a small decrease (two- to threefold) in AMP MIC (0.06 µg/mL for COM15Δ*pbp5*) vs MIC (0.12–0.19 µg/mL for its parental wild type) (Table 1); the small decrease is consistent with our earlier observation that this strain produces very little if any PBP5 (16). On the other hand, another clade B strain TX1330, previously shown to produce more PBP5 than WT COM15 in western blots (16), showed a greater reduction in AMP MIC when its *pbp5* was deleted (from AMP MIC 1–1.5 µg/mL for WT to 0.01–0.09 µg/mL for Δ*pbp5*) (Table 1). The *in situ* reconstituted strain, TX1330*Δpbp5::pbp5*_TX1330_, showed an AMP MIC similar to that with WT TX1330 (Table 1). The AMP MIC of the published clade A1 partial *pbp5* deletion mutant E1162Δ*pbp5* was 0.25 µg/mL (24); when reconstituted *in situ* with *pbp5* from the parental E1162, AMP MICs were 32 µg/mL, similar to the WT E1162 (Table 1).

The plasmid construct designs and MIC results are shown in Fig. 1 and Tables 2 to 5, respectively. To evaluate the effect on AMP MICs generated by *pbp5* alleles from strains belonging to different clades and that show different AMP MICs and different proportions of “S” amino acids and “R” amino acids, we cloned *pbp5* alleles into the previously published vector pCWR620 (*ftsW*_C68_/*psr*_C68_); this vector (kindly provided by LB Rice and derived from pTCV lac) has *ftsW* and *psr* from C68, a (sub)clade A1, AMP-R strain (15). The AMP MICs of the host strain *E. faecium* D344SRF and the various plasmid constructs and WT strains are shown in Table 2. The AMP MICs of D344SRF harboring the empty vector pTCV-lac showed no change vs D344SRF alone (0.023 µg/mL) (Tables 2 and 3). *E. faecium* D344SRF derivatives harboring pCWR620 *pbp5-R* alleles from AMP-R, clade A1 strains were all highly AMP-R, with MICs similar to the respective WT strains that were the source of the *pbp5* allele, namely C68 (AMP 128 µg/mL), 1.231.502 (AMP 128–256 µg/mL), and TX82 (AMP 64 µg/mL) and one clade A2 strain E1679 (AMP 256 µg/mL) (Table 2).

**TABLE 5:** Influence of host background on AMP MICs generated by *pbp5* alleles previously generated (12) to express higher levels of AMP resistance

Constructs used (*pbp5* allele and clade)	AMP MICs (µg/mL)	References
*E. faecium* COM15 wild type (clade B)	0.12–0.19^*a*^	(10, 14, 20)
COM15Δ*pbp5*	0.064	This study
COM15Δ*pbp5* (pCWR624^*b*^)	3–4	This study
COM15Δ*pbp5* (pCWR666^*c*^)	3–4	This study
*E. faecium* TX1330 wild type (clade B)	1–1.5	(16, 20 )
TX1330Δ*pbp5*	0.01–0.09	This study
TX1330Δ*pbp5* (pCWR624)	3–4	This study
TX1330Δ*pbp5* (pCWR666)	2–3	This study
*E. faecium* E1162 wild type (clade A1)	24–32	(24)
E1162Δ*pbp5*	0.12–0.19	(24)
E1162Δ*pbp5* (pCWR624)	128–256	This study
E1162Δ*pbp5* (pCWR666)	128–256	This study

^*a*^ Range of MICs reflects results with colonies and/or repetitions.

^*b*^ pCWR624 kindly provided by LB R 47ice is pCWR620 with *pbp5* (S7/R14) from clade A2 strain D366 mutagenized to contain methionine, isoleucine, and glutamic acid at positions 485, 499, and 629 in the active site of PBP5. No serine at position 466 (15).

^*c*^ pCWR666 kindly provided by LB Rice is pCWR620 with *pbp5* from clade A1 strain C68 (S1/R20) previously mutagenized to contain serine at 466 position in addition to having methionine, isoleucine, and glutamic acid at positions 485, 499, and 629 in the active site of PBP5 (15).

On the other hand, D344SRF harboring pCWR620 plasmid with *pbp5* alleles from strains with lower AMP MICs (E980 and COM15) showed moderate AMP MICs ranging from 3 to 8 µg/mL. The differences between AMP MICs generated with *pbp5-R* alleles vs *pbp5-S* and *pbp5-R/S* alleles would be expected (15) and reflect the greater number of those amino acids previously associated with and/or experimentally shown to influence AMP MICs (14, 15, 18). While the AMP MICs generated by the constructs with *pbp5-S* and *pbp5-R/S* alleles were somewhat higher than the AMP MICs of the respective WT strains from which these alleles originated, this is likely due to plasmid copy number generating more copies of PBP5.

### Influence of *ftsW/psr* from different clades on AMP MICs generated by various naturally occurring *pbp5* alleles cloned in *trans* into *E. faecium* D344SRF

Since pCWR620 (15) used above contains *ftsW*/*psr* from a clade A1, AMP-R strain (C68), we next investigated the influence of *ftsW*/*psr* from a clade B, AMP-S strain on AMP MICs generated by the different *pbp5* alleles (Table 3). To achieve this, we cloned *ftsW*/*psr* from COM15 (clade B; AMP MIC 0.12–0.19 µg/mL) into pTCV-lac resulting in pTEX6162 and then inserted different *pbp5-R* alleles into this vector, as above. The resulting derivatives with four different *pbp5-R* alleles showed considerably lower AMP MICs (range 4–16 µg/mL) than their WT parent clade A1 strains [C68, 1.231.502, TX82, and clade A2 strain E1679 (AMP MIC generally >64 µg/mL)] (Table 3) and lower MICs than constructs with these *pbp5* alleles cloned into the same vector backbone (pCWR620) downstream of the clade A *ftsW_C68_*/*psr_C68_* (AMP MIC range, 64–256 µg/mL). We also inserted *pbp5-S* alleles from three different AMP-S strains into pTEX6162 (*ftsW*_COM15_/*psr*_COM15_). The resulting strains showed considerably lower MIC than when these alleles were downstream of the clade A *ftsW_C68_*/*psr_C68_* in pCWR620 (Table 3). Also, as above, while slightly increased AMPs (MIC range of 0.5–3 µg/mL) were seen vs their respective WT parent strains (Table 3), this is likely due to plasmid copy number.

To look further at the roles of *psr* and *ftsW*, we generated additional plasmid constructs derived from pTCV-lac with *psr-*only from each clade (without *ftsW*) and hybrid constructs with *ftsW* from one clade and *psr* from the other. AMP MICs for derivatives of D344SRF with these plasmid constructs and various *pbp5* alleles (Table 4) showed that AMP MICs were low with clade A1 *ftsW*_C68_ plus clade B *psr*_COM15_ (third column, Table 4) as well as with clade B *psr*_COM15_ alone (second column, Table 4) and were approximately equal to those when both *psr* and *ftsW* were from clade B (last column, Table 3). On the other hand, with the clade B *ftsW*_COM15_ plus the clade A1 *psr*_C68_ hybrid construct (last column Table 4) as well as with clade A1 *psr*_C68_ alone (first column, Table 4), AMP MICs generated by the different *pbp5* alleles were much higher and approximately equal to AMP MICs seen when both *psr* and *ftsW* were from clade A (middle column, Table 3).

We next explored the effect on AMP MICs when plasmids pCWR624 and pCWR666 [both of which have clade A1 *ftsW_A1_/psr_A1_* from C68 with downstream *pbp5* alleles previously engineered (15) to express high levels of AMP-R] were introduced into strains deleted for *pbp5* but which retained their own *psr* and *ftsW* (Table 5). In contrast to the high AMP MICs seen when these plasmids were introduced into D344SRF (a clade A1 strain lacking *pbp5*, *psr*, and *ftsW*), AMP MICs were low (2–4 µg/mL) when introduced into each of the clade B strains (COM15Δ*pbp5* and TX1330Δ*pbp5*). One possible explanation for this difference is that the presence of the endogenous clade B *psr* results in greater susceptibility to AMP regardless of the presence of *psr*_A1_; however, an effect of some other component of the clade B host background is certainly possible. In contrast, as expected, high MICs (128–256 µg/mL) resulted when these plasmids were introduced into the clade A1 strain E1162*Δpbp5* (Table 5) with both a *cis* and a *trans* copy of *psr*_A1_.

### FtsW amino acid sequence alignments

The comparison of FtsW amino acid sequence alignments revealed an 8/387 amino acid difference between clade A1 and clade B strains. Within the subset of clade A1 strains examined, C68, TX82, and E1162 shared a 100% identical sequence, whereas TX16 exhibited a variance of 1/387 amino acids, and TX1.231.502 had an 11/387 amino acid deletion. Among clade B strains (COM15, E980, TX1330), there were only 3/387 amino acids that differed. These findings collectively indicate a generally high conservation of the FtsW amino acid sequence (Fig. S2).

### Quantitative real-time PCR

**Fig 2:**
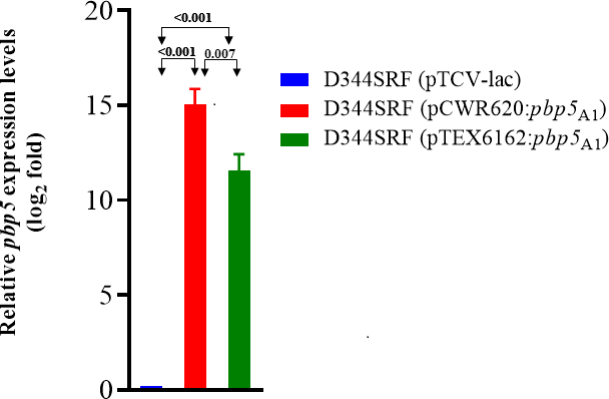
Quantitative real-time PCR for *pbp5* expression. Comparative expression levels (log_2_ fold) of the *pbp5*_A1_ gene from strain TX82 cloned into strain D344SRF using pTCV-lac (empty vector), pCWR620 which has *ftsW*_A1_/*psr*_A1_ from clade A1 *Efm* strain C68 upstream of *pbp5*_TX82_, and pTEX6162 which has *ftsW*_B_/*psr*_B_ from clade B strain COM15. Three biological replicates per test bacteria were used, and for each biological replicate, three technical replicates were used.

We next determined *pbp5* transcript levels when clade A1 *pbp5* from TX82 was cloned into pCWR620 (with clade A1 *ftsW*_C68_/*psr*_C68_) and when the same *pbp5-R* was cloned into pTEX6162 (with clade B *ftsW*_COM15_/*psr*_COM15_) relative to empty vector control pTCV-lac. Each construct was in strain D344SRF (lacking *pbp5*/*psr/ftsW* and other surrounding gene/s). With pCWR620 with *pbp5-R*, the *pbp5* transcription level was 15.04 (log_2_ fold) vs 11.55 (log2 fold) with pTEX6162 (Fig. 2); this 1.3 log_2_ fold difference was statistically significant (*P* 0.007). These results indicate that the lower AMP MICs conferred by *pbp5-R* in the presence of clade B *psr* vs the high AMP MICs in the presence of clade A *psr* are at least in part due to lower *pbp5* transcript levels.

### PBP5 detection by western blotting

**Fig 3:**
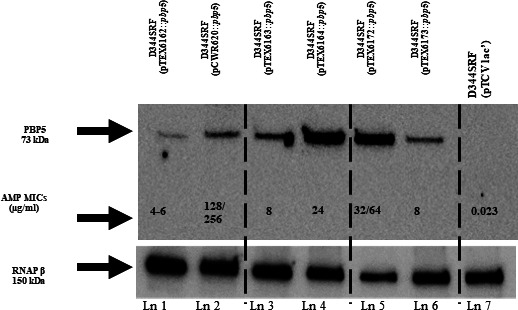
Differential expression of PBP5 from clade A1 strain TX82 with *ftsW ± psr* genes from different clades. The western blot contains results using different *ftsW ± psr* constructs with *pbp5* from clade A1 TX82 (S0/R21) cloned into pTCV-lac vector in the host *E. faecium* strain D344SRF. Ampicillin (AMP) MICs for each construct are given per each lane as described in Tables 2 to 4. *ftsW* and *psr* genes with subscript B are from COM15; *ftsW* and *psr* genes with subscript A1 are from C68. *pbp5* is from TX82. Total protein samples of equal quantity underwent separation on 4%–20% Protein Gels and transferred onto a nitrocellulose membrane. PBP5 was identified using a polyclonal serum (pre-absorbed with D344SRF to remove non-specific bands) targeting rPBP5-R from various constructs. Below the blots, loading control probed with a monoclonal antibody to RNA polymerase β subunit. Lane 1 (pTEX6162 = *ftsW_B_*/*psr_B_::pbp5*), lane 2 (pCWR620 = *ftsW*_A1_/*psr*_A1_::*pbp5*), lane 3 (pTEX6163 = *ftsW*_A1_/*psr*_B_::*pbp5*), lane 4 (pTEX6164 = *ftsW*_B_/*psr*_A1_::*pbp5*), lane 5 (pTEX6172 = *psr*_A1_::*pbp5*), lane 6 (pTEX6173 = *psr*_B_::*pbp5*), and lane 7 D344SRF (pTCV-lac).

The impact of *ftsW*/*psr* from different clades on cloned *pbp5* (from TX82, clade A) in *trans* into *E. faecium* D344SRF was assessed through western blotting. Analysis using a polyclonal serum raised against recombinant PBP5-R (rPBP5-R) from strain C68 revealed higher PBP5 protein levels in plasmids carrying the clade A *psr*_C68_, irrespective of the *ftsW* origin or presence (Fig. 3). This observation again suggests that *ftsW* does not contribute to the observed increase in PBP5 levels on western blots. Likewise, TX82 *pbp5* (S0/R21) in plasmids containing *psr*_COM15_ exhibited low PBP5 levels, regardless of the *ftsW* origin or presence, also supporting that *ftsW* appears not to influence the PBP5 levels or MICs detected in western blots (Fig. 3). The MICs of constructs without *ftsW* displayed MICs equal to or at most slightly lower than those with *ftsW* (32–64 µg/mL vs 128–256 µg/mL for clade A gene(s) and 4–6 µg/mL vs 8 µg/mL for clade B strains) (Tables 3 and 4; Fig. 3). These differences are within the error expected in MIC.

We also observed that in the case of *pbp5* cloned downstream of *ftsW*_C68_/*psr*_C68_ (lane 2, Fig. 3) with AMP MIC 64/128, the western blot analysis revealed a comparatively lower PBP5 presence than that observed with the same *pbp5* cloned, in the same vector background, downstream of *ftsW*_COM15_/*psr*_C68_ or *psr*_C68_ (lanes 4, 5, Fig. 3) despite these later constructs generating lower AMP MICs (24 and 32 µg/mL, respectively). No discernible differences in the levels of the RNA polymerase β subunit were noted except in lane 5 which might have occurred due to unknown/technical reason.

The lack of a strict correlation between protein levels seen with *pbp5* with *ftsW*_C68_/*psr*_C68_ (lane 2, Fig. 3) vs the same *pbp5* with *ftsW*_COM15_/*psr*_C68_ or *psr*_C68_ (lanes 4, 5, Fig. 3) is not particularly surprising, as posttranscriptional, translational, and posttranslational regulatory networks are known to influence protein abundance (16, 35). Previous studies have demonstrated instances where genes with similar mRNA levels exhibited up to a 20-fold difference in protein abundance (36). In our prior research involving a separate published study, there was not complete agreement observed between PBP5 protein levels, *pbp5* mRNA levels, and their corresponding AMP MICs (16, 35). It is also conceivable that hybrid *ftsW/psr* combinations may in some unknown way impact the AMP MICs, perhaps via an interaction with the PBP5 target proteins by *ftsW*_C68_/*psr*_C68_, which exhibited a higher AMP MIC range.

## DISCUSSION

Historically, high-level AMP resistance in *Efm* has been attributed primarily to amino acid sequence changes in PBP5 (11, 14, 15, 37). It has been demonstrated that specific *pbp5* alleles are associated with specific *Efm* clades and that some specific substitutions found in clade A1 PBP5s result in a decrease in the affinity of this protein for penicillin and in higher AMP MICs (16). However, *pbp5* sequence variation alone has not been able to explain the range of MICs seen in *Efm* (11, 14), and expression differences have also been demonstrated (16). The region upstream of *pbp5* has been suggested to contribute to levels of AMP resistance (17), although a direct role of the upstream Psr in *pbp5* expression has been debated (12, 15). Previously, we reported evidence of considerable variance in the *ftsW/psr* region immediately upstream of the *pbp5* gene, including DNA fragment insertions and deletions in this region, among *Efm* strains from different clades and with different ranges of AMP MICs; these differences generally appeared to correlate with AMP MICs and the differential abundance of PBP5, with clade A strains consistently having a truncated or interrupted *psr* relative to *psr* from clade B strains (16).

The results presented in this study agree with prior observations that allele differences in *pbp5* are important for AMP MICs. We show the effect of various naturally occurring *pbp5* alleles from clades A1, A2, and B on the AMP MIC when cloned and introduced in *trans* into *Efm* strain D344SRF, lacking the region encompassing *ftsW*, *psr*, and *pbp5*. D344SRF derivatives harboring *pbp5* alleles from AMP-R clade A1 strains cloned into a vector with upstream clade A1 *fstW/psr* were all highly AMP-R, with MICs similar to the respective WT strains (Table 2). On the other hand, D344SRF with the same vector containing cloned *pbp5* alleles from AMP-S or intermediate clade B and A2 strains showed low to moderate levels of AMP MICs (Table 2). We also noted that plasmids with *pbp5-S* and -*R/S* alleles resulted in higher AMP MICs than seen with their respective WT strain. This could be due to plasmid copy number resulting in the production of more PBP5, the impact of the upstream clade A1 *psr* or *ftsW* in the vector, or other host factors. Zhang et al., for example, reported additional genes within *Efm* genomes that contribute to elevated ampicillin MICs (24).

Most importantly, our results point to the importance of *psr* in modulating AMP resistance in *Efm*. Specifically, the origin and genomic context/sequence of the *ftsW/psr* region were shown to be important for variations seen among AMP MICs (Tables 3 and 4). By cloning combinations of *ftsW ± psr* from different clades together with distinct *pbp5-S*, *R/S*, or *R* alleles into D344SRF (which lacks its own *ftsW/psr/pbp5*), we have shown that the presence of *psr*_B_ leads to lower expression of PBP5 and lower MICs compared to the presence of *psr*_A_, regardless of which *pbp5* allele is present. Specifically, in the presence of clade A1 *psr* [pCWR620 (*ftsW*_C68_*/psr*_C68_)] and different *pbp5* alleles, the AMP MICs were considerably higher (Tables 2 and 3) than in the presence of clade B *psr* [pTEX6162 (*ftsW*_COM15_*/psr*_COM15_)]. As shown in Table 4, having *ftsW* from a differing clade than *psr* had little effect on the AMP MICs. Moreover, *psr*_B_ and *psr*_A_ cloned alone with their own promoter, without *ftsW*, resulted in the same phenotype as *psr* cloned with an *ftsW* allele (Table 4). These results indicate that *psr* alone can account for the differences in AMP MIC seen with the *ftsW/psr* hybrid clones and that *ftsW* does not appear to play a considerable role under these conditions. Western blot analysis of PBP5 expression not only confirmed the influence of *psr* on PBP5 but also suggested that *ftsW* is not a significant contributing factor to the observed protein level variation (Fig. 3).

When considering the implications of these results, one consideration is that it might be possible to generate compounds to mimic a Psr_B_ effect to decrease PBP5-R expression and lower Amp MICs as a possible avenue for new antimicrobials. Whether AMP MICs of WT clade A1 strains which have a WT-truncated *psr*_A_ would be lowered by a Psr_B_-mimic is not clear, as we were unable to successfully introduce stable plasmids containing *psr*_B_ into WT clade A1 strains that contain their endogenous *ftsW/psr/pbp5*. However, we were able to show (Table 5) that a clade A1-truncated *psr*_A_ (present in the pCWR plasmids as *ftsW*_C68_*/psr*_C68_*/pbp5*_C68_) does not lead to high MICs when placed in the clade B strains COM15Δ*pbp5* and TX1330Δ*pbp,* which still have their chromosomal *psr*_B_, i.e., the presence of clade A *psr* did not overcome an effect of the presence of clade B *psr.* If other host background influences were to be ruled out, this would imply that Psr_B_ is “dominant” to Psr_A_ since the presence of both A and B *psr* alleles (Table 5) in derivatives of COM15Δ*pbp5* and TX1330Δ*pbp5* still resulted in low MICs.

The mechanism for the influence of Psr on *pbp5* expression and AMP MICs is yet to be determined. Psr (renamed LcpA for *E. hirae* Psr) belongs to the LytR-CpsA-Psr family (38). This family of proteins is found in most gram-positive bacteria, and some contain more than one Lcp protein (39). This protein family is poorly understood, and a multitude of functions has been reported, including cell surface properties, virulence, antibiotic resistance, and septum formation (40–43). Structural and domain analysis of Psr sequences revealed the presence of an HTH domain and a transmembrane domain, likely as domains associated with transcriptional attenuation. However, in the present study, we did not perform any experiments showing that Psr acts as repressor protein or regulates the PBP5 expression by binding to an operator DNA sequence. This remains a subject for our future studies. In *E. hirae*, evidence suggests that LcpA plays a role in cell wall metabolism, “probably acting as a phosphotransferase catalyzing the attachment of cell wall polymers to the peptidoglycan” (40). Thus, the mechanism by which Psr_B_ lowers AMP MICs and PBP5 expression may be indirect, perhaps by a cell wall metabolism effect resulting in feedback repression of PBP5 expression, while truncated and presumably inactive Psr_A_ does not. Such a phenomenon is not without precedent since it has been reported in other organisms that the nature of the peptidoglycan precursors can alter the level of β-lactam resistance associated with the expression of low-affinity PBPs (44–46).

Overall, the results presented in this study provide further insight into ampicillin resistance of *E. faecium* clade A strains, highlighting that, in addition to amino acid sequence alterations in PBP5, the *psr* region is important for the expression of PBP5 and AMP resistance. The presence of full-length *psr*_B_ results in lower expression of PBP5 and lower MICs than does the presence of a shorter *psr*_A_, regardless of the *pbp5* allele present (Table 4). Lower AMP MICs were also seen when *psr* alleles from each clade were present together in clade B strains, although an effect of other differences between clade A and B strains may also contribute. It is interesting that, in the split between clade A and clade B *E. faecium* and the further evolution of higher AMP MICs in hospital-associated strains, changes in *pbp5* appear to have co-evolved with changes in *psr* that appear to have resulted in an inactive Psr, allowing higher levels of expression together with more ampicillin-resistant PBP5. Further studies are warranted in order to elucidate the underlying mechanism for the differential regulation of PBP5 and ampicillin resistance by *psr* between the *Efm* clades.

## SUPPLEMENTAL MATERIAL

Supplemental material mbio.00170-24-s0001.docx Supplemental tables and figures.

ASM does not own the copyrights to Supplemental Material that may be linked to, or accessed through, an article. The authors have granted ASM a non-exclusive, world-wide license to publish the Supplemental Material files. Please contact the corresponding author directly for reuse.
