# Supplementary material for: Genomic context as well as sequence of both psr and penicillin-binding protein 5 contributes to β-lactam resistance in Enterococcus faecium
Source: mBio. 2024 Apr 2;15(5):e00170-24. doi: 10.1128/mbio.00170-24 (PMC11077988; doi:10.1128/mbio.00170-24)
Supplement: Supplemental material — Supplemental tables and figures. [file mbio.00170-24-s0001.docx]

**Supplementary Material**

**Supplementary Table S1.** Bacterial strains and plasmids used in the study.

| **Strains and**  **plasmids** | **Relevant characteristic(s) including clades, PBP5 amino acids profile**  **(PBP5-S/R) and isolation source.** | **Reference(s)** |
| --- | --- | --- |
| ***E. faecium*** |  |  |
| D344SRF | Host strain for the *pbp5* constructs. Parent strain was a hospital-associated  clinical *E. faecium* that was AMP resistant (24 mcg/ml) prior to a 160 kb  spontaneous genomic deletion encompassing *pbp5*, *ftsW, psr* regions.  AMP MIC 0.023 µg/ml. | (15, 18) |
| COM15 | Fecal isolate from healthy volunteer. Clade B, PBP5 amino acid profile  S21/R0. AMP MIC 0.12 – 0.19 µg/ml). | (10, 14, 20) |
| E980 | Fecal isolate from healthy volunteer. Clade B, PBP5 amino acid profile  S16/R5. AMP MIC 0.75 –1 µg/ml. | (14, 20, 21) |
| D366 | Urine isolate (human). Clade A2, PBP5 amino acid profile S7/R14.  AMP MIC 1.5 µg/ml). | (11, 14) |
| E1679 | Vascular catheter tip (human). Clade A2, PBP5 amino acid profile S3/R18.  AMP MIC 192 - 256 µg/ml. | (21) |
| C68 | Fecal isolate from hospitalized patient. Clade A1, PBP5 amino acid profile  S1/R20. AMP 128 µg/ml. | (16, 22) |
| 1.231.502 | Blood isolate from hospitalized patient. Clade A1, PBP5 amino acid profile  S1/R20. AMP >256 µg/ml. | (16, 22) |
| TX82 | Blood isolate from patient with endocarditis. Clade A1, PBP5 amino acid  profile S0/R21. AMP 64 µg/ml. | (16, 23) |
| E1162 | Clinical isolate, bloodstream, Clade A1, PBP5 amino acid profile S_1_/R_20_.  AMP 24 – 32 µg/ml. | (24) |
| ***E. faecium***  **constructs** |  |  |
| TX6153 | COM15Δ*pbp5*, markerless deletion mutant. | This study |
| TX6159 | COM15Δ*psr*, markerless deletion mutant. Deleted intragenic 456/885 bp of  *psr* coding region. | This study |
| TX6260 | TX1330Δ*pbp5*, markerless deletion mutant. | This study |
| TX6262 | TX1330Δ*pbp5*::*pbp5*_TX1330_ reconstituted *in situ* in the chromosome. | This study |
| TX2205 | E1162Δ*pbp5* mutant. We determined via sequencing that this mutant has  truncated *pbp5*, i.e., 567 bp/2037 bp of *pbp5* from start codon are present  while remaining *pbp5* coding region, i.e., 1470/2037 bp was deleted. | (24) |
| TX6261 | E1162Δ*pbp5*::*pbp5*_E1162_ reconstituted *in situ* in the chromosome.  Complemented fragment encompassed 66 bp upstream of  *pbp5* start codon (includes promoter region) + 2037 bp of *pbp5* coding  region + 629 bp downstream of *pbp5* sequence. | This study |
| TX6286 | COM15Δ*pbp5* (pCWR624) | This study |
| TX6287 | COM15Δ*pbp5* (pCWR666) | This study |
| TX6288 | TX1330Δ*pbp5* (pCWR624) | This study |
| TX6289 | TX1330Δ*pbp5* (pCWR666) | This study |
| TX6290 | E1162Δ*pbp5* (pCWR624) | This study |
| TX6291 | E1162Δ*pbp5* (pCWR666) | This study |
| TX6269 | D344SRF(pTEX6162::*pbp5*S21/R0_COM15), consists *pbp5* and upstream promoter. | This study |
| TX6231 | D344SRF(pTEX6162::*pbp5*S16/R5_E980) consists *pbp5* and upstream promoter. | This study |
| TX6230 | D344SRF(pTEX6162::*pbp5*S7/R14_D366) consists *pbp5* and upstream promoter. | This study |
| TX6228 | D344SRF(pTEX6162::*pbp5*S3/R18_E1679) consists *pbp5* and upstream promoter. | This study |
| TX6225 | D344SRF(pTEX6162::*pbp5*_S1/R20_C68_) consists *pbp5* and upstream promoter. | This study |
| TX6226 | D344SRF(pTEX6162::*pbp5*S1/R20_1.231.502) consists *pbp5* and upstream promoter. | This study |
| TX6227 | D344SRF(pTEX6162::*pbp5*S0/R21_TX82) consists *pbp5* and upstream promoter. | This study |
| TX6259 | D344SRF(pCWR620::*pbp5*S21/R0_COM15) consists *pbp5* and upstream promoter. | This study |
| TX6258 | D344SRF(pCWR620::*pbp5*S16/R5_E980) consists *pbp5* and upstream promoter. | This study |
| TX6257 | D344SRF(pCWR620::*pbp5*S7/R14_D366) consists *pbp5* and upstream promoter. | This study |
| TX6256 | D344SRF(pCWR620::*pbp5* S3/R18_E1679) consists *pbp5* and upstream promoter. | This study |
| TX6253 | D344SRF(pCWR620::*pbp5*S1/R20_C68) consists *pbp5* and upstream promoter. | This study |
| TX6254 | D344SRF(pCWR620::*pbp5*S1/R20_1.231.502) consists *pbp5* and upstream promoter. | This study |
| TX6255 | D344SRF(pCWR620::*pbp5*S0/R21_TX82) consists *pbp5* and upstream promoter. | This study |
| TX6237 | D344SRF(pTEX6163::*pbp5*S21/R0_COM15) consists *pbp5* and upstream promoter. | This study |
| TX6238 | D344SRF(pTEX6163::*pbp5*S16/R5_E980) consists *pbp5* and upstream promoter. | This study |
| TX6235 | D344SRF(pTEX6163::*pbp5*S7/R14_D366) consists *pbp5* and upstream promoter. | This study |
| TX6234 | D344SRF(pTEX6163::*pbp5*S3/R18_E1679) consists *pbp5* and upstream promoter. | This study |
| TX6232 | D344SRF(pTEX6163::*pbp5*S1/R20_C68) consists *pbp5* and upstream promoter. | This study |
| TX6233 | D344SRF(pTEX6163::*pbp5*S1/R20_1.231.502) consists *pbp5* and upstream promoter. | This study |
| TX6236 | D344SRF(pTEX6163::*pbp5*_S0/R21_TX82_) consists *pbp5* and upstream promoter. | This study |
| TX6244 | D344SRF(pTEX6164::*pbp5*S21/R0_COM15) consists *pbp5* and upstream promoter. | This study |
| TX6245 | D344SRF(pTEX6164::*pbp5*S16/R5_E980) consists *pbp5* and upstream promoter. | This study |
| TX6242 | D344SRF(pTEX6164::*pbp5*S7/R14_D366) consists *pbp5* and upstream promoter. | This study |
| TX6241 | D344SRF(pTEX6164::*pbp5*S3/R18_E1679) consists *pbp5* and upstream promoter. | This study |
| TX6239 | D344SRF(pTEX6164::*pbp5*S1/R20_C68) consists *pbp5* and upstream promoter. | This study |
| TX6240 | D344SRF(pTEX6164::*pbp5*S1/R20_1.231.502) consists *pbp5* and upstream promoter. | This study |
| TX6243 | D344SRF(pTEX6164::*pbp5*_S0/R21_TX82_) consists *pbp5* and upstream promoter. | This study |
| TX6265 | D344SRF(pTEX6172::*pbp5*S21/R0_COM15) consists *pbp5* and upstream promoter. | This study |
| TX6266 | D344SRF(pTEX6172::*pbp5*S16/R5_E980) consists *pbp5* and upstream promoter. | This study |
| TX6267 | D344SRF(pTEX6172::*pbp5*S7/R14_D366) consists *pbp5* and upstream promoter. | This study |
| TX6268 | D344SRF(pTEX6172::*pbp5*S1/R20_C68) consists *pbp5* and upstream promoter. | This study |
| TX6269 | D344SRF(pTEX6172::*pbp5*S1/R20_1.231.502) consists *pbp5* and upstream promoter. | This study |
| TX6270 | D344SRF(pTEX6172::*pbp5*_S0/R21_TX82_) consists *pbp5* and upstream promoter. | This study |
| TX6271 | D344SRF(pTEX6173::*pbp5*S21/R0_COM15) consists *pbp5* and upstream promoter. | This study |
| TX6272 | D344SRF(pTEX6173::*pbp5*S16/R5_E980) consists *pbp5* and upstream promoter. | This study |
| TX6273 | D344SRF(pTEX6173::*pbp5*S7/R14_D366) consists *pbp5* and upstream promoter. | This study |
| TX6274 | D344SRF(pTEX6173::*pbp5*S1/R20_C68) consists *pbp5* and upstream promoter. | This study |
| TX6275 | D344SRF(pTEX6173::*pbp5*S1/R20_1.231.502) consists *pbp5* and upstream promoter. | This study |
| TX6276 | D344SRF(pTEX6173::*pbp5*_S0/R21_TX82_) consists *pbp5* and upstream promoter. | This study |
| TX6251 | D344SRF(pTEX6165::*pbp5*S21/R0_COM15) consists *pbp5* and upstream promoter. | This study |
| TX6252 | D344SRF(pTEX6165::*pbp5*S16/R5_E980) consists *pbp5* and upstream promoter. | This study |
| TX6249 | D344SRF(pTEX6165::*pbp5*S7/R14_D366) consists *pbp5* and upstream promoter. | This study |
| TX6248 | D344SRF(pTEX6165::*pbp5*S3/R18_E1679) consists *pbp5* and upstream promoter. | This study |
| TX6246 | D344SRF(pTEX6165::*pbp5*S1/R20_C68) consists *pbp5* and upstream promoter. | This study |
| TX6247 | D344SRF(pTEX6165::*pbp5*S1/R20_1.231.502) consists *pbp5* and upstream promoter. | This study |
| TX6250 | D344SRF(pTEX6165::*pbp5*_S0/R21_TX82_) consists *pbp5* and upstream promoter. | This study |
| **Plasmids** |  |  |
| Blunt II-TOP | Plasmid used for initial cloning of PCR fragments; KAN^r^. | Thermo  Fisher™ |
| pTCV-lac | Shuttle vector pTCV-lac, KAN ^r^. | (31) |
| pHOU1 | Conjugative donor plasmid that carries GEN^r^ and the counterselectable  pheS* gene. | (26-28) |
| pCWR620 | pTCV-lac with *ftsW* and *psr* from *E. faecium* strain C68 (clade A1) extending  from upstream of the *ftsW_Efm_* promoter to downstream of the *pbp5* promoter  cloned into the SmaI/BamHI site of shuttle vector, KAN^r^. | (15) |
| pTEX6162 | pTCV-lac with *ftsW* and *psr* derived from *E. faecium* COM15 (clade B)  extending from upstream (~300 bp upstream of the *ftsW* promoter to  downstream of the full *psr,* intergenic region between *psr* and *pbp5* plus  putative conserved -10 and -35 regions (among clade A and clade B  *E. faecium*)) of *pbp5* cloned into the SmaI/BamHI site of shuttle vector, KAN^r^. | This study |
| pTEX6163 | pTCV-lac with *ftsW*_C68_ and *psr*_COM15_. Same strategy used as for pTEX6162,  encompassing from upstream of the *ftsW*_C68_ promoters to downstream of the  *psr*_COM15_ cloned into the SmaI/BamHI site of shuttle vector, KAN^r^. | This study |
| pTEX6164 | pTCV-lac with *ftsW*_COM15_ and *psr*_C68_. Same strategy used as for pTEX6162,  encompassing from upstream of the *ftsW*_COM15_ promoters to downstream of  the *psr*_C68_ cloned into the SmaI/BamHI site of shuttle vector, KAN^r^. | This study |
| pTEX6165 | pTCV-lac with *ftsW*_1.231.502_ and *psr*_COM15_. Same strategy used as for pTEX6162,  extending from upstream of the *ftsW*_1.231.502_ promoters to downstream of the  *psr*_COM15_ cloned into the SmaI/BamHI site of shuttle vector, KAN^r^. | This study |
| pTEX6172 | pTCV-lac with *psr*_C68_ (clade A1). AMP MIC results seen with various *pbp5*  alleles cloned in vectors pTEX6162– pTEX6165 appeared to suggest that there  may be some influence of *psr* on *pbp5* expression and resulting AMP MICs,  therefore, we created this vector to study this. Here, 74 nucleotides extending  of upstream of the *psr*_C68_ start codon to 21 nucleotides downstream of the stop  codon (intergenic region between *psr* and *pbp5*) cloned into the SmaI/BamHI  site of shuttle vector, KAN^r^. Both regions upstream and downstream  are conserved in C68 (A1) and COM15 (clade B) and the same set of primers  worked for both strains to amplify the *psr* fragments. | This study |
| pTEX6173 | pTCV-lac with *psr*_COM15_ (clade B). Created for same reasons as explained for  pTEX6172.Here, 74 nucleotides extending of upstream of the *psr*_COM15_ start  codon to 67 nucleotides downstream of the stop codon (intergenic region  between *psr* and *pbp5*) cloned into the SmaI/BamHI site of shuttle vector, KAN^r^. | This study |
| pCWR624 | pTCV-lac with *ftsW* and *psr* from strain C68 (A1) to assess the contributions of different individual amino acid mutations to expression of AMP resistance in *E. faecium*. Amino acids residues at positions 485, 499 and 629 in the active region of PBP5 are methionine, isoleucine and glutamic acid. No serine at 466 position. | (15) |
| pCWR666 | pTCV-lac with *ftsW* and *psr* from strain C68 (A1) to assess the contributions of different individual amino acid mutations to expression of AMP resistance in *E. faecium*. Amino acids residues at positions 485, 499 and 629 in the active region of PBP5 are alanine, threonine and valine, respectively, in addition to having serine at 466 position. | (15) |
| ***E coli*** | DH5α *E coli* host strain used for routine cloning. | Invitrogen |

**Supplementary Table S2.** Oligonucleotides used in the study.

| **Primer Name** | **Sequence 5’- 3’ and purpose** | **Restriction-**  **sites underlined** |
| --- | --- | --- |
| **TX6153** | **For COM15Δ*pbp5* construct** |  |
| psr-F_UP | CGGGATCCCATGGATTTTCGTCTGC | BamHI |
| pbp5-R_UP | AGGTATTGCGCCTGTTCGATTTTTGCCGTGCTTGTC |  |
| pbp5-3p-F_DW | CGAACAGGCGCAATACCTCAACCAAAATTATCAATAA |  |
| CPA2-R2 | CGGAATTCCTGGATTGAATTGGGTC | EcoRI |
| **TX6159** | **For COM15Δ*psr* construct** |  |
| Psr UP-F #3 | ATTTGCGGCCGCCTGGCTTCCCCAGATGTTCA | NotI |
| Psr UP-R #1 | CCAGCTTCTACTGCTTGGGT |  |
| Psr DW-F #4 | AGACGAAAATTACCGAAAGCGGTGGGAAAA |  |
| Psr DW-R #7 | CGGGATCCGTTGCTTCTTGCGTGGTCAG | BamHI |
| **TX6260** | **For TX1330Δ*pbp5* construct** |  |
| UP #62 | ATTTGCGGCCGCCAGATTCACTTATGGTTGC | NotI |
| UP #65 | GAAGTCGTTCTTTTGCTTGTTATTC |  |
| DW #66 | CAAGCAAAAAGAACGACTTCATAACTATAG |  |
| DW #61 | CGGGATCCCAGAAAAAGTGTACGCAAATTCTTC | BamHI |
| **TX6262** | **For TX1330Δ*pbp5*::*pbp5*_TX1330_ reconstituted in chromosome** |  |
| #67 | ATTTGGCCGCGATCGATACATTATCCGTACCAG | NotI |
| #61 | CGGGATCCCAGAAAAAGTGTACGCAAATTCTTC | BamHI |
| **TX6261** | **For E1162Δ*pbp5*::*pbp5*:_E1162_ reconstituted in chromosome** |  |
| # 51 | ATTTGCGGCCGCTATGTTAGAATAAACAGGTATAAATAGTG | NotI |
| # 52 | CGGGATCCCAGAAAAAGTGTACGCAAATTCTTC | BamHI |
| **TX6227** | **For pTEX6162, i.e., pTCV-lac::*ftsW*_COM15_/*psr*_COM15_** |  |
| FtsW, #104F | GCGCCCGGGCCTTGTTAGGTGAGGCTCCTATAC | SmaI |
| #131R | CTTTCATTGCGAATTCCTCTTTC |  |
| Psr, # 130F | GAAAGAGGAATTCGCAATGAAAG |  |
| #105R-New | TGCGGATCCCACTATTTATACCTGTTTATTCTAAC | BamHI |
|  | **For pTEX6172, i.e., pTCV-lac:*:psr*_C68_ and pTEX6173, i.e., pTCV-lac:*:psr*_COM15_ vectors** |  |
| #136-F+ #105R-New | GCGCCCGGGCTCAGTTTGAATTATCATAAAGTGCGGATCCCACTATTTATACCTGTTTATTCTAAC | SmaI site |
|  | **For various *pbp5* amplification and cloning into vectors pCWR620, pTEX6162, pTEX6163,**  **pTEX6164, pTEX6165, pTEX6172 and pTEX6173 at BAMHI site** |  |
| #135-F +  #100 R new | TGCGGATCCAAAATAAAGGAATAACAAGC + TGCGGATCCTTATTGATAATTTTGGTTGAGG to generate *pbp5* from COM15 | BamHI |
| #133-F +  #100 R new | TGCGGATCCAAAATAAAGGAATAGCAAGC, to generate *pbp5* from E980 | BamHI |
| #134-F +  #100 R new | TGCGGATCCAAAATAAAGGAATGACAAGC, to generate *pbp5* from D366 | BamHI |
| #132-F +  #100 R New | TGCGGATCCAAATAAAGGAATGACAAGC to generate *pbp5* from E1679, C68, 1.231.502 and TX82 | BamHI |
| #137 F1-*pbp5* | ACGGGATCTCACAAGAAGATTAC, for  qRT PCR |  |
| #138-R1-*pbp5* | AACGTAGATCCAGGAGCATAAC for  qRT PCR |  |
| #148 F1-*gyr* | AGCTCGTCAATGTGCTACTG for  qRT PCR |  |
| #149-R1-*gyr* | CATATCGCGGACTGCTTCTT for  qRT PCR |  |

**Supplementary Figure S1.** Schematic representation of the *pbp5* and surrounding genes of clade B *E. faecium* strains COM15 and subclade A1 C68 (16)a.


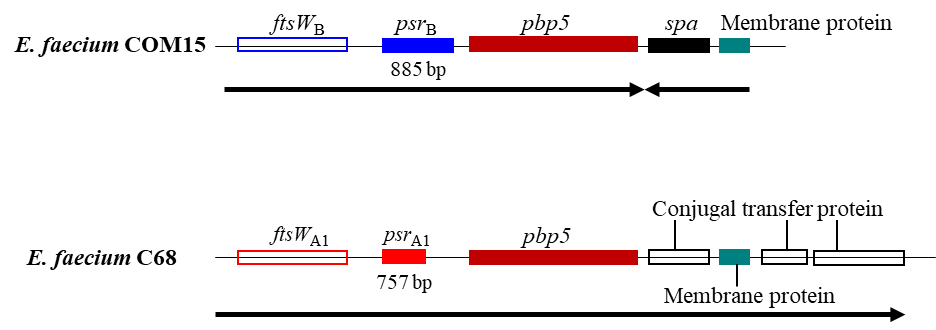


*ftsW*B *- filamenting temperature sensitive.*

*psr - pbp5 synthesis repressor* (full length, 885 bp*)* from clade B *E. faecium* strain in blue and truncated (757 bp) with premature stop codon is from *E. faecium* C68, sub clade A1, is in red.

*pbp5 - penicillin-binding protein 5.*

*spa - Sodium proton antiporter.*

a = Data were derived from reference (16).

**Supplementary Figure S2.** Alignment of FtsW amino acids in *E. faecium* strains associated with clades A1, A2, and B.

**Clade B**

**Clade A1**

**Clade A2**
